# Supplementary material for: Immediate action is the best strategy when facing uncertain climate change
Source: Nat Commun. 2018 Jul 2;9:2566. doi: 10.1038/s41467-018-04968-1 (PMC6028488; doi:10.1038/s41467-018-04968-1)
Supplement: Supplementary file 1 — Supplementary Information [file 41467_2018_4968_MOESM1_ESM.pdf]

Supplementary Information: Facing uncertain climate change, immediate action is the best strategy

Maria Abou Chakra[1,2], Silke Bumann[1], Hanna Schenk[1], Andreas Oschlies[3], and Arne Traulsen[1]

[1] Department of Evolutionary Theory, Max-Planck-Institute for Evolutionary Biology, August-Thienemann-Straße 2, 24306 Plön, Germany

[2] University of Toronto, Donnelly Centre for Cellular and Biomolecular Research, 160 College Street, Toronto M5S 3E1, Canada

[3] Biogeochemical Modeling, GEOMAR Helmholtz Centre for Ocean Research Kiel, Düsternbrooker Weg 20, 24105 Kiel, Germany

April 27, 2018

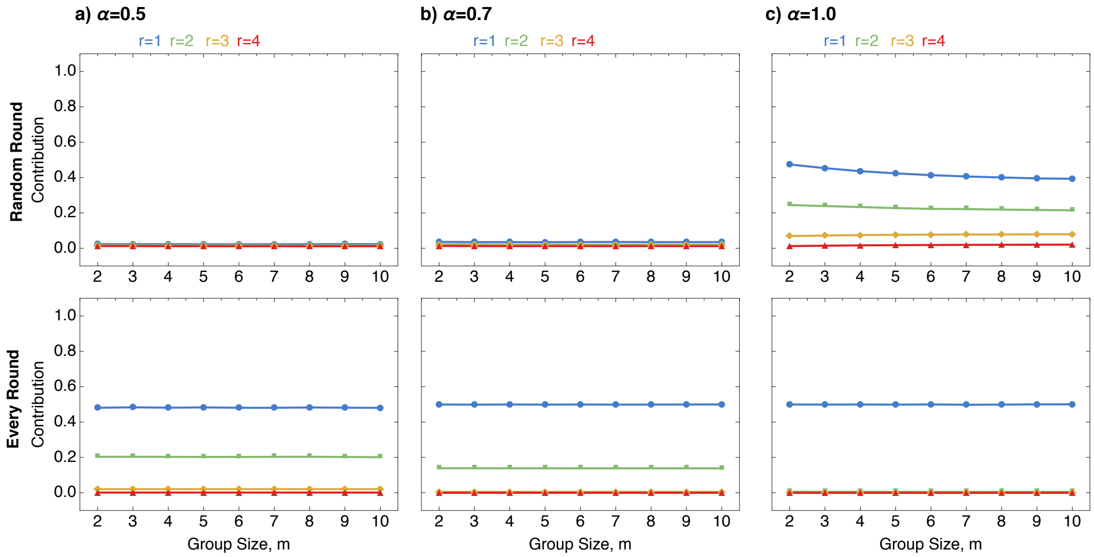

Supplementary Figure 1: Simulations for a 4 round game showing the average individual contributions as the number of rich players changes within a group. Upper panels show a single loss in a random round, lower panels a loss in every round. For $\alpha = 0.5$ (a) and $\alpha = 0.7$ (b), contributions only occur if there are multiple losses and they mostly occur on the first round. When losses are complete, $\alpha = 1$ in c), players contribute just enough in the first round to avoid further losses in the case of multiple losses. For a single loss, they tend to contribute slightly later in the game (Risk curve $p[C_r] = \left(1 + \exp\left[10\left(\frac{C_r}{W_0} - \frac{1}{2}\right)\right]\right)^{-1}$, parameters: population size $N = 100$, 1000 games per generation, mutation rate $\mu = 0.03$, and the standard deviation for mutations in the individual decision thresholds τ_r is set to $\sigma = 0.15$)

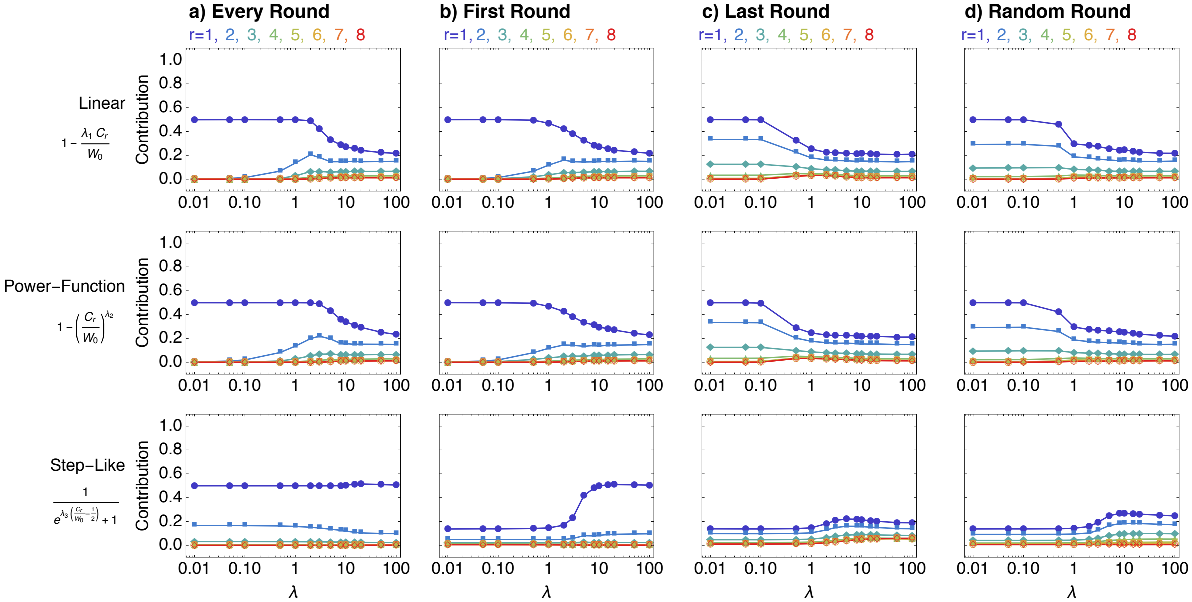

Supplementary Figure 2: Contributions for different timings of potential losses in an eight round game. The graphs depict average contributions in each round of an eight-round game with a potential loss in (a) every round, (b) the first round, (c) the last or (d) a random round. It is assumed that individuals lose everything, i.e. $\alpha = 1$. A noticeable trend across all timings is that most contributions are made in the first round (blue line) (games are pair-wise, initial wealth of both players $W_0 = 2$, population size $N = 100$, 1000 games per generation, mutation rate $\mu = 0.03$, and the standard deviation for mutations in the individual decision thresholds τ_r is set to $\sigma = 0.15$).

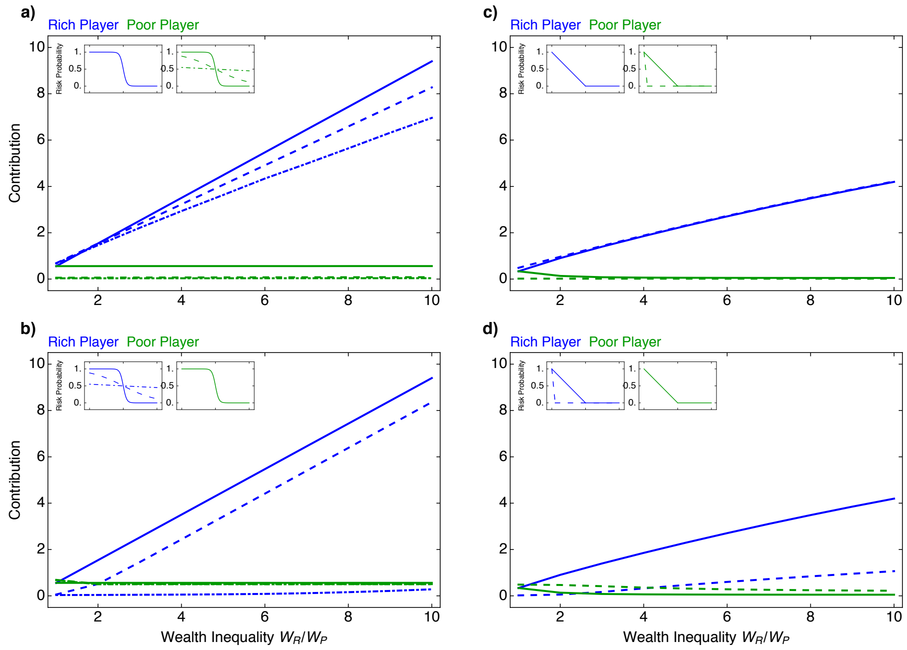

Supplementary Figure 3: Wealth and risk heterogeneity. In a single round game with two players, the contributions of the rich player increase as wealth inequality between the rich and the poor players increases. Without wealth inequality, $W_{\mathrm{R}}/W_{\mathrm{P}} = 1$, the contributions are the same. Using $p = (1 + \exp\left[\lambda(\frac{C}{W} - \frac{1}{2}\right])^{-1}$, we see that the rich player contributes even when the risk is varied between the rich and poor,(a) here we fixed the risk curve of the rich player $\lambda_{\mathrm{R}} = 1$ and varied the risk of the poor player by changing λ_{P} from 1 to 0.1 and 10. (b) here we fixed the risk curve of the poor player $\lambda_{\mathrm{P}} = 1$ and varied the risk of the rich player by changing λ_{R} from 1 to 0.1 and 10. Using $p = 1 - \lambda C/W$, we see the same trend as in (a) and (b) . (c) here we fixed the risk curve of the rich player $\lambda_{\mathrm{R}} = 1$ and decreased the risk of the poor player by changing λ_{P} from 1 to 10. (d) here we fixed the risk curve of the poor player $\lambda_{\mathrm{P}} = 1$ and decreased the risk of the rich player by changing λ_{R} from 1 to 10 (parameters $\Omega = 1$, $m = 2$, $W_{\mathrm{P}} = 1$, $W_{\mathrm{R}} \in [1, 10]$).

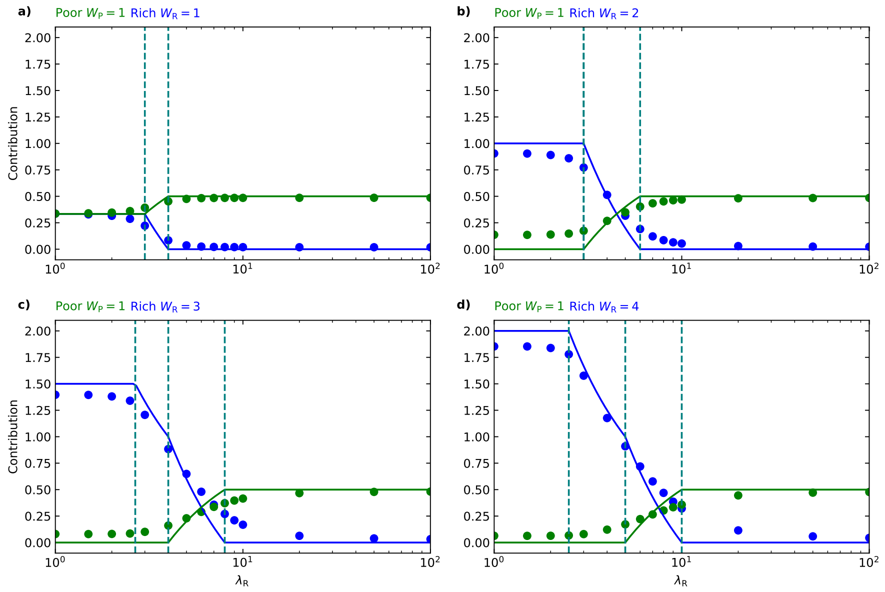

Supplementary Figure 4: Contributions of rich and poor players for different wealth inequalities. In general, a rich player contributes more when her wealth increases. With decreasing risk for the rich (increasing λ_{R}), rich players start investing less. Eventually, the poor start contributing to compensate for the declining contribution of the rich. a) shows a scenario where the initial wealth of both players is identical, $W_{\mathrm{R}} = W_{\mathrm{P}} = 1$. In b), the rich player has twice the wealth of the poor player, $W_{\mathrm{R}} = 2W_{\mathrm{P}}$. in c) three times ($W_{\mathrm{R}} = 3W_{\mathrm{P}}$) and in d) four times ($W_{\mathrm{R}} = 4W_{\mathrm{P}}$). Analytical results according to Table 1 in the main text are represented by lines, simulations as dots. As the simulations include randomness from finite population size and finite selection strength, some deviations between them and the analytical results (which implicitly assume infinite population size and no randomness) are expected (pairwise games, $m = 2$, with one round $\Omega = 1$, loss fraction $\alpha_{\mathrm{R}} = \alpha_{\mathrm{P}} = 1$, constant initial wealth for the poor $W_{\mathrm{P}} = 1$ but varying W_{R} and linear risk curves with the risk of the rich equal to (when $\lambda_{\mathrm{R}} = 1$) or lower than the risk for the poor).
